# Novel Antifungal Compounds, Spermine-Like and Short Cyclic Polylactates, Produced by *Lactobacillus harbinensis K.V9.3.1Np* in Yogurt

**DOI:** 10.3389/fmicb.2018.02252

**Published:** 2018-10-09

**Authors:** Amor Mosbah, Emilie Delavenne, Yasmine Souissi, Mouna Mahjoubi, Philippe Jéhan, Nicolas Le Yondre, Ameur Cherif, Arnaud Bondon, Jérôme Mounier, Michèle Baudy-Floc’h, Gwenaelle Le Blay

**Affiliations:** ^1^CNRS, ISCR–UMR 6226, Université de Rennes 1, Rennes, France; ^2^BVBGR-LR11ES31, ISBST, Université de la Manouba, Biotechpole Sidi Thabet, Ariana, Tunisia; ^3^UEB, EA3882, SFR148 ScInBioS, ESIAB, Technopôle Brest-Iroise, Plouzané, France; ^4^Laboratoire Universitaire de Biodiversité et Ecologie Microbienne, Université de Brest, Plouzané, France; ^5^Univ Rennes, CNRS, ScanMAT - UMS 2001, Rennes, France; ^6^Laboratoire des Sciences de l’Environnement Marin, UMR 6539, Université de Brest, Université Européenne de Bretagne, Institut Universitaire Européen de la Mer, Plouzané, France

**Keywords:** spermine, polylactates, lactobacillus, extraction, purification, Ms, Nmr

## Abstract

*Lactobacillus harbinensis K.V9.3.1Np* was described as endowed with high antifungal activity. Most of the studies associated this activity to the produced organic acids, i.e., lactic acid, acetic acid, and hexanoic acid. The aim of this study was to purify and identify, other not yet described, antifungal molecules produced by *L. harbinensis K.V9.3.1Np* when used in yogurt fermentation. Active compounds were extracted through several extraction processes using organic solvents and protein precipitation. The fractions of interest were purified using flash chromatography and preparative HPLC for specific characterization. The bioactive compounds identification was performed using Nuclear Magnetic Resonance and Mass Spectrometry. Activity tests against *Penicillium expansum* and *Yarrowia lipolytica* showed that the active compounds from *L. harbinensis K.V9.3.1Np* are benzoic acid and a polyamine identified as a spermine analog, which has not been reported earlier. However, the highest activity was shown by a mixture of short (*n* = 2–5) polycyclic lactates. Our overall results demonstrate the efficiency of the proposed extraction/purification approach. The new compounds described here have promising antifungal activities but further studies are still needed to decipher their mode of action and production pathways. Even though, they present an interesting potential application in food, feed, as well as, in pharmaceutical industries and could serve as alternative to chemical additives.

## Introduction

There is a growing concern on microbiological safety of dairy food as their contamination can cause considerable economic losses. More specifically, the contamination of food matrices with various mycotoxins or other undesirable fungal metabolites became a serious issue in food industry (Moretti et al., 2017). Molds’ development in food may be accidental or related to environmental factors promoting fungal growth and metabolite secretion. The fungal contamination inducing physicochemical deterioration of dairy foods is usually concomitant to organoleptic damages which consist mainly in off-odors and flavors associated to visible color and texture defects. In fermented milk products, fungal growth induces a decrease in the acidity promoting subsequently the growth of some undesirable microorganisms (Leyva Salas et al., 2017). Thus, the need of preventing food from microbiological spoilage challenged scientists to develop appropriate preservative solutions. Avoiding microbial contamination is achieved through suitable treatment, and/or appropriate preservative agents or by preventing the growth and the activity of those that were not eliminated after treatment (Leyva Salas et al., 2017).

Legislations and consumers requirements have led food manufacturers to look for an alternative to chemical preservatives and additives. In this context, the use of bioprotective agents is considered as a suitable solution to alleviate fungal deterioration. Biopreservation is ensured by natural or controlled microbiota and/or their metabolites. Among the most suitable candidates as natural preservative agents, Gram-positive lactic acid bacteria (LAB) are widely used (Dalié et al., 2010; Voulgari et al., 2010; Crowley et al., 2013). Historically, four main “core genera” were defined being involved in food fermentation (*Lactobacillus*, *Leuconostoc*, *Pediococcus*, and *Streptococcus*) (Wessels et al., 2004). However, a reclassification was achieved and several other genera were added to this original grouping, including *Lactococcus*, *Micrococcus*, *Oenococcus*, *Aerococcus* and others (Crowley et al., 2013). Homofermentative and heterofermentative LAB induce pH decrease and produce antimicrobial molecules generating naturally conserved fermented foods. The antimicrobial mechanisms associated to the LAB involve a wide range of metabolite production including organic acids, phenyllactic acid, reuterin, cyclic dipeptides, fatty acids as well as proteinaceous and miscellaneous antifungal compounds (Crowley et al., 2013). Moreover, LAB have been granted a GRAS (Generally Recognized As Safe) status and were also included to the “Qualified Presumption of Safety” (QPS) list in Europe (Crowley et al., 2013; Delavenne et al., 2013).

Recently, a new strain isolated from cow milk and identified as *Lactobacillus harbinensis K.V9.3.1Np*, demonstrated a strong antifungal activity against six species commonly involved in yogurt fungal spoilage, *Debaryomyces hansenii*, *Rhodotorula mucilaginosa*, *Yarrowia lipolytica*, *Penicillium brevicompactum*, *Kluyveromyces lactis*, and *Kluyveromyces marxianus* (Delavenne et al., 2015). In fermented yogurt, the antifungal activity was not affected through the storage and the technological processes indicating its adequacy as a substitute to chemical additives (Delavenne et al., 2013). In separate reports, Belguesmia et al. (2014) and Mieszkin et al. (2017) quantified and tested several organic acids produced by *L. harbinensis K.V9.3.1Np*, such as acetic, lactic, hexanoic, 2-hydroxybenzoic, and 2-pyrrolidone-5-carboxylic acids. These compounds were shown to constitute part of the overall antifungal activity of the strain by acting in synergy with other, yet unidentified antimicrobial agents.

The aim of the current study was to identify, purify and characterize potential new compounds responsible for the antifungal activity of *L. harbinensis K.V9.3.1Np*. Our experimental design was based on a multi-step extraction and purification protocols (proteins and organic fractions) from 7 liters-yogurt culture. The identified biomolecules were then subjected to structural elucidation using nuclear magnetic resonance spectroscopy and mass spectrometry.

## Materials and Methods

### Microorganisms and Culture Conditions

*Lactobacillus harbinensis K.V9.3.1Np* was previously isolated from cow milk (Delavenne et al., 2012). It showed strong antifungal activity against a panel of yeasts and molds in yogurt as an antifungal protective culture (Delavenne et al., 2013).

Commercialized freeze-dried yogurt starters (« ferments lyophilisés pour yaourt brassé », Nat-Ali, Nantes, France), containing *Streptococcus salivarius* subsp. *thermophilus* (*S. thermophilus*) and *L. delbrueckii* subsp. *bulgaricus* (*L. bulgaricus*) were used for yogurts manufacture. These two strains were also isolated from this commercialized freeze-dried yogurt starters and identified by sequencing of the rpoA (*L. bulgaricus*) and 16S rRNA (*S. thermophilus*) genes.

Lactobacilli and *S. thermophilus* were stored in MRS (AES Chemunex, Bruz, France) and M17, respectively, supplemented with glycerol (30%, v/v) at -80°C. They were routinely cultivated in MRS (lactobacilli) or M17 supplemented with lactose 0.5% (*S. thermophilus*) at 30°C for *L. harbinensis* and 37°C for *L. bulgaricus* and *S. thermophilus*.

*Yarrowia lipolytica* UBOCC 211004 and *Penicillium expansum* UBOCC 108102, two fungi commonly encountered in dairy food and yogurt spoilage (Fleet, 1990; Cousin, 2002), and particularly resistant to organic acids (Delavenne et al., 2013), were chosen as indicators for antifungal assays. They came from the Culture Collection of Université de Bretagne Occidentale (UBOCC, Plouzané, France). *P. expansum* was cultivated on potato dextrose agar (PDA, Difco, Le Pont de Claix, France) slants for few days at 25°C until spores were formed. Spores were then harvested with sterile distilled water supplemented with 0.1% Tween-80 and the suspension was spread on PDA in a Roux flask to increase spore production (Roy et al., 1996). Spores were harvested, enumerated using a Malassez cell and stock suspensions were standardized to a final concentration of 10^7^ spores/ml before storage at -80°C in glycerol (10%, v/v).

*Yarrowia lipolytica* was stored in yeast extract and malt based medium (YEMA) supplemented with glycerol (30%, v/v) at -80°C, and cultivated aerobically at 25°C on YEMA agar.

### Yogurt Supernatant Recovering, Activity Control, and Protein Precipitation

Yogurts were produced with half-fat pasteurized milk supplemented with low-fat milk powder (4%) (Lait en poudre écrémé, Casino, France). After heating at 85°C for 30 min, milk was rapidly cooled to 45°C before being inoculated with the milk freeze-dried starter according to the manufacturer instruction. Milk was then mixed and dispatched as 100 ml cultures into glass containers of 120 ml.

Preliminary control experiments and activity detection were performed on 1 L-cultures inoculated or not with *L. harbinensis* at a final concentration of 5.10^7^ CFU/ml. Subsequent experiments aiming to separate and purify protein and organic fractions were then achieved with 7 L-culture inoculated with *L. harbinensis*. Fermentations were conducted at 42°C for 6 h. Yogurts were stored for 2 weeks at for 10°C after fermentation. Yogurts were centrifuged for 10 min at 14.000 *g* and supernatant was recovered by filtration on 0.45-μm nitrocellulose filter.

From the 7-L inoculated culture, protein precipitation was achieved using the following procedure (modified from Bhattacharyya and Babu (2009): (i) Ammonium sulfate precipitation (from 10% saturation to 100% saturation in 10 steps) by gradual incorporation of the salt and stirring for 2 h at 4°C and centrifugation at 9000 *g* for 20 min for each step. The precipitate of each step was resuspended in water at 4°C; (ii) From each recovered fraction (*n* = 10), soluble proteins with low molecular mass and peptides were separated by cell centricon (Amicon Millipore), with 10 kDa cut-off. A total of 20 FSP fractions were collected, lyophilized and tested for their antifungal activity against the indicator strains.

### Solvent Extraction and Purification of Secondary Metabolites

Ethyl acetate extractions and activity tests were performed on the 1 L inoculated and non-inoculated control cultures and on the 7 L inoculated culture. From this later, protein-free supernatant was extracted with the same volume of ethyl acetate (three times) (Khan et al., 2016). The organic phases were combined and evaporated to dryness, under vacuum at 45°C. The resulting DOA fraction (3.4 g) was solubilized in ethyl acetate dried with anhydrous MgSO_4_ generating a major soluble fraction named FOP (3 g) and a minor insoluble fraction resuspended in bidistilled water FOE (0.4 g).

The first step of the purification of the organic ethyl acetate fraction (FOP) was assessed by flash chromatography on silica gel (Stevens and Hill, 2009). Elution was performed using dichloromethane (DCM)-methanol gradient (0 to 10% methanol for 18 min followed by an isocratic flow of 10% methanol for 10 min and finally isocratic gradient of 20% methanol-80% DCM for 20 min). The final purification was achieved by TLC with DCM-methanol (90:10, by vol.) and spot visualized in UV detector. A total of 18 fractions were evaporated under vacuum at 45°C and stored at -20°C until used for antimicrobial tests (Uzair et al., 2018).

### Chromatography Purification

Active FOP and FOE fractions were analyzed by RP-HPLC on a XTerra C18 column (4.6 mm^∗^100 mm, 3.5 mm) using a Waters 2696 system equipped with a 600 PDA and Empower software. Solvents A and B were H_2_O and MeCN supplemented with 0.08% (v/v) TFA. Linear 0–60% gradients of B into A over 45 min, 60–95% B into A for1 min followedby 95% A isocratic gradient during 10 min were used for elution at 1 mL/min flow rate, with UV detection at 214, 268, 280, and 220 nm. For separation, preparative RP-HPLC was performed usingan XTerra C18 column (19 mm × 300 mm, 10 mm, Waters) with the solvents A and B, 0.1% TFA (v/v) in H_2_O and 0.08% TFA (v/v) in MeCN, respectively. For elution we used linear 0–60% gradients of B into A over 40 min, 60–95% B into A for1 min followedby an isocratic gradient during 10 min with 95% A and 5% B at 10 mL/min flow rate, with UV detection at 214 nm (Solecki et al., 2015).

### Antifungal Activity of Fractions

The antifungal activity of each fraction was determined by the agar diffusion method (Delavenne et al., 2015). Yeast suspensions were prepared in sterile 0.1% peptone water by scraping colonies from the surface of YEMA agar after 2 days incubation at 25°C. Spores suspensions were directly prepared from the stock suspensions. Cells and spores were enumerated with a Malassez cell. Appropriate amount of spores or yeasts cells suspension were added to YEMA agar (0.8%) to achieve a final concentration of 5.10^4^ spores or cells/ml. The medium was mixed properly and poured in Petri dishes (90 mm). After solidification, wells of 9 mm diameter were made in the center of each agar plate. FSP fractions were dissolved in 200 μl water/methanol 80/20 and FOP fractions in 200 μl of DCM/methanol 80/20. Each well of both *Y. lipolytica* and *P. expansum* agar plates, were filled with 80 μl of the tested fractions. Water/methanol 80/20 and DCM/methanol 80/20 were both used as negative controls. Agar plates were then put at 10°C for 2 h to allow products diffusion, and then incubated at 25°C for 48 to 72 h. Upon incubation time, inhibition zones were measured.

### NMR Analysis

1D NMR spectra were recorded using a Bruker Avance 500 spectrometer equipped with a BBO cryoprobe at the PRISM core facility (Rennes, France). Sample were analyzed in CDCl_3_ solution or in water solution with 10% D_2_O using a sequence with presaturation.

### ESI-TOF, ESI-MS/MS, and LC-ESI-Q-TOF Analysis

All ms Spectra were recorded at on Waters Q-TOF 2 and Bruker MicrOTOF-Q II and Maxis 4G Mass Spectrometers the CRMPO core facility (Rennes, France).

## Results

Several studies were conducted using a targeted approach on the antifungal activity of *L. harbinensis K.V9.3.1Np* to assess the active biomolecules (Belguesmia et al., 2014; Mieszkin et al., 2017). The obtained results suggested that acetic, lactic, hexanoic 2-hydroxybenzoic, and 2-pyrrolidone-5-carboxylic acids, acted as antifungal compounds but other antifungal agents, not targeted in these studies, could be involved in the high antifungal potential. In the current work, using an untargeted approach, we focused on the identification, purification, and characterization of all non-acids antifungal compounds produced by *L. harbinensis*.

### Screening of the Fractions Harboring Antifungal Activity

Antifungal susceptibility tests of the different extracts were carried out using the disk diffusion method. After the fermentation process with *L*. *bulgaricus*, S. *thermophilus* amended or not (control culture) with *L. harbinensis K.V9.3.1Np*, the yogurt culture supernatant was separated into different fractions using ammonium sulfate precipitation, ethyl acetate, and dichloromethane. Then the extracted fractions were tested using *P. expansum* and *Y. lipolytica* as reference organisms. Antifungal activity was retrieved only from the organic extracts of the *L. harbinensis* inoculated cultures (data not shown).

**FIGURE 1:**
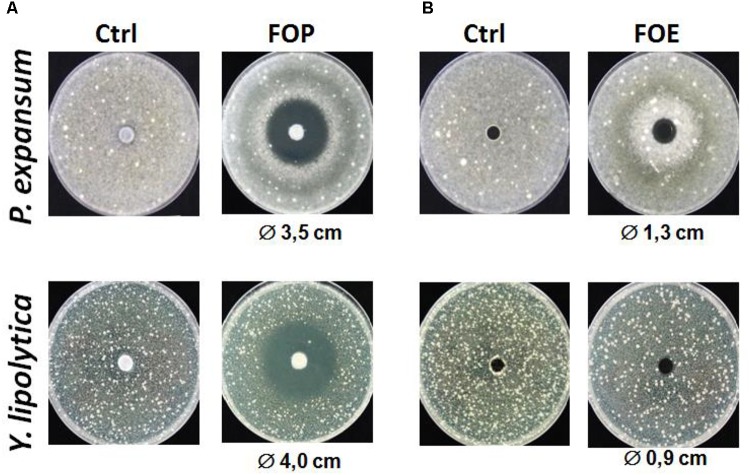
Antifungal activity of *Lactobacillus harbinensis K.V9.3.1Np* ethyl acetate extracts against *Penicillium expansum* and *Yarrowia lipolytica*. **(A)** Antifungal activity of the organic extract (FOP) with the control DCM/methanol 80/20 (Ctrl); and **(B)** Antifungal activity of the aqueous extract (FOE) respect to the control water/methanol 80/20 (Ctrl).

In a second step, the organic and aqueous extracts using ethyl acetate and dichloromethane were tested. As preliminary results shown in **Figure 1**, we observed a quite pronounced inhibition zone for the organic fraction of *L. harbinensis K.V9.3.1Np* supernatant extracted with ethyl acetate (FOP). In fact, after 48 h of incubation at 25°C with the disks, clearing zones of 3.5 mm and 4 cm in diameter were observed in *P. expansum* and *Y. lipolytica*, respectively (**Figure 1A**). The aqueous fraction (FOE) also exhibited an antifungal activity but of a relative weak intensity compared to the organic one (**Figure 1B**). Based on these results, we focused on the ethyl acetate organic extracts for further characterization of *L. harbinensis K.V9.3.1Np* anti-fungal potential.

### Identification of Bioactive Extracts

Ethyl acetate organic fraction (FOP) is a mixture of several compounds that may react in an antagonistic or synergistic way to exhibit the overall antifungal activity. Thus, we were interested in the identification of the bioactive compounds present in this fraction. To rapidly target the molecule responsible for this activity, a flash chromatography was performed. This technique was used to increase the compounds separation resolution and to collect individually purified molecules.

**FIGURE 2:**
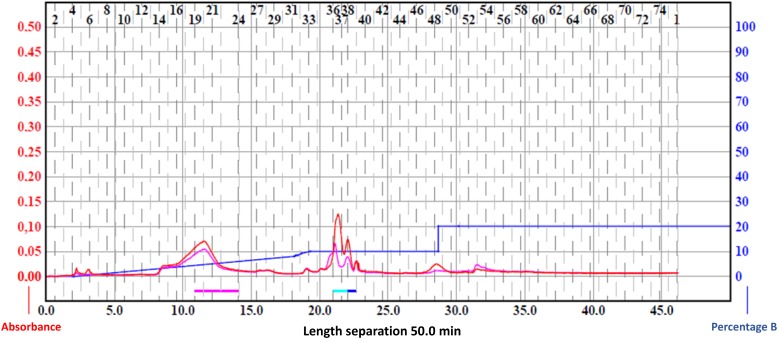
Chromatogram of the ethyl acetate fractions using flash chromatography.

**FIGURE 3:**
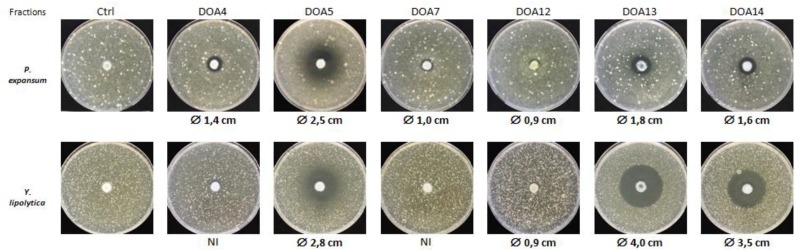
Antifungal activity of the most active fractions obtained by flash chromatography against *P. expansum* and *Y. lipolytica*.

Eighteen fractions were collected (**Figure 2**) and then dried to be used for antifungal assays using *P. expansum* and *Y. lipolytica* indicator strains. Among the tested fractions, the active ones are presented in **Figure 3**. The inhibition zone varied with the different sub-extracts as well as with the two tested fungal strains. Six fractions, i.e., DOA4, DOA5, DOA7, DOA12, DOA13, and DOA14, showed an interesting inhibition zone ranging from 0.9 to 4 cm.

### Selection of Sub-Fractions of Interest From the Identified Bioactive Extracts

**FIGURE 4:**
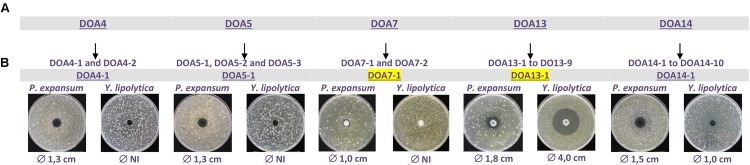
Antifungal activities of HPLC purified DOA4-1, DOA5-1, DOA7-1, DOA13-1, and DOA14 against *P. expansum* and *Y. lipolytica*.

Further investigation was performed on the antifungal activity of these collected fractions. For that purpose, an additional fractionation step was performed using preparative chromatography. The results shown in **Figure 4A** clearly demonstrated that those fractions contained multiple compounds that may be responsible for this antifungal activity. Thus, a second activity test was achieved with the recovered sub-fractions. After this step, we could identify sub-fractions DO4-1, DO5-1, DO7-1, DO13-1, and DO14-1 as responsible for this antifungal activity (**Figure 4B**).

### Compounds Identification in the Sub-Fractions of Interest

**FIGURE 5:**
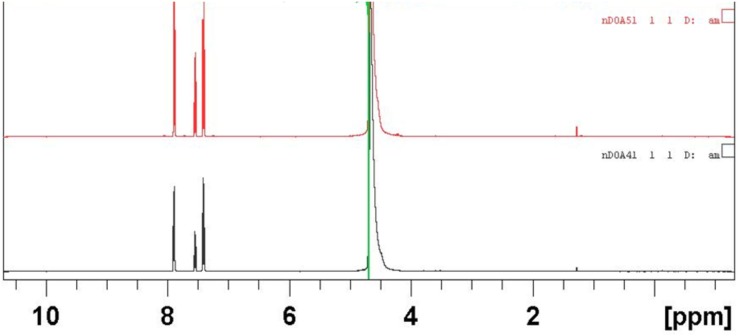
NMR spectra in water of the actives compounds identified as benzoic acid in the DOA4 and DOA5 fractions.

**FIGURE 6:**
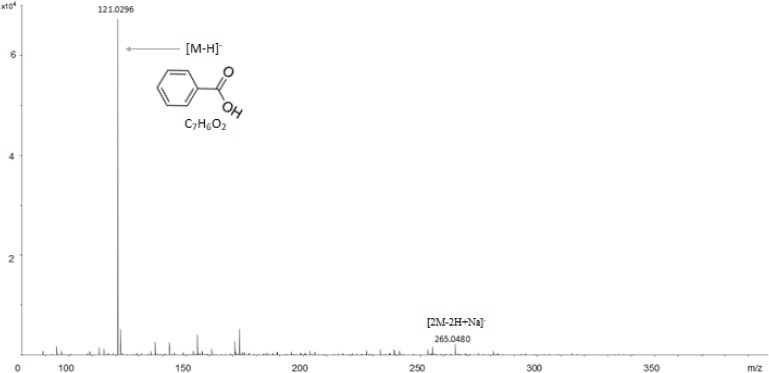
ESI-TOF analysis of the actives compounds identified as benzoic acid in the DOA4 and DOA5 fractions.

The structural elucidation of the compounds present in sub-fractions DO4-1, DO5-1, DO7-1, DO13-1, and DO14-1 was a key step of this study. Thus, mass spectrometry and nuclear magnetic resonance were used for that purpose. Structural elucidation of both compounds DOA4-1 and DOA5-1 was based on NMR spectral analysis. These compounds were identified as benzoic acid as clearly shown in **Figure 5**. In addition, **Figure 6** shows their mass spectra obtained by ESI-TOF and confirmed their identification as benzoic acid.

**FIGURE 7:**
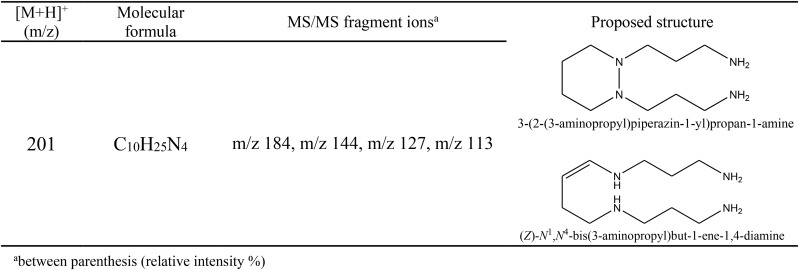
MS profile of the DOA7-1 active compound obtained using a Waters Q-TOF II Mass Spectrometer. The proposed structures are unsaturated polyamine named spermine analogous.

The purified fraction DOA7-1 also exhibited an interesting antifungal activity. ESI-MS/MS was performed in order to elucidate the structure of this compound. Based on the fragment ions observed after collision induced dissociation associated to the molecular formula assigned to these ions using high resolution measurement, two structures were proposed for the compound DOA7-1 (**Figure 7**). The proposed structures were unsaturated polyamine named spermine analogous compounds that have not been reporter earlier.

It is worth noting that the attributions of these structures were initially based on the difference of 2 mass unit with spermine (precursor ion [MH]^+^ m/z 203). Moreover, the molecular formula attributed to the DOA7-1 molecule with high resolution measurement was C_10_H_24_N_4_ (experimental mass: 201.2082, theoretical mass: 201.2079, and relative error: 1 ppm). The spermine molecular formula being C_10_H_26_N_4_, it is irrevocable that a cyclization occurs or an instauration is added to form the new structure explaining the loss of the two hydrogen. Moreover, for all the amines, in MS/MS analysis, the loss of a neutral fragment of ammonia led to the formation of a fragment with an m/z signal that corresponded to the species [M+H-NH_3_]^+^. According to literature for spermidine, the product ion was generated by the loss of 2 molecules of ammonia, giving m/z 112 [M+H-2NH_3_]^+^ (Sagratini et al., 2012). For spermine, the fragmentation of the precursor ion [MH]^+^ m/z 203, produced an ion at m/z 112 [M+H-(CH_2_)_3_N_2_H_4_-NH_3_]^+^ (Sagratini et al., 2012). These results are coherent with the proposed structures as we observed after MS/MS fragmentation of the precursor ion [MH]^+^ m/z 201, a product ion m/z 184 [M+H-NH_3_]^+^. The other characteristic product ions confirmed further the proposed structures as they corresponded to specific losses associated to this structure. In fact, the ions m/z 144, m/z 127, m/z 113 were identified as [M+H-(CH_2_)_3_NH_3_]^+^, [M+H-NH_3_-(CH_2_)_3_NH_2_]^+^, and [M+H-(CH_2_)_3_NH_2_-(CH_2_)NH_3_]^+^.

**FIGURE 8:**
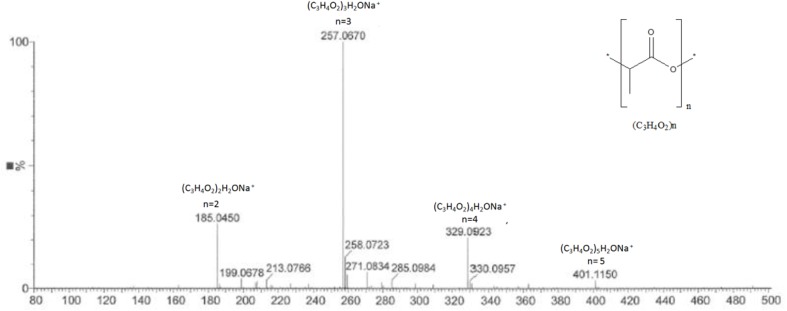
MS profile of DOA13-1 and DOA14 active compounds obtained using Bruker Maxis 4G Mass Spectrometer II Mass Spectrometer. The short polycondensation of lactic acid to form a short poly (lactic acid) is described in equation (1).

The analysis of the most active compound DOA13-1 using Maxis 4G II, LC-ESI-Q-TOF, revealed the presence of polyester compounds probably formed through the polycondensation of lactic acid molecule. The short polycondensation of lactic acid to form a short poly (lactic acid) is described in equation (1). In fact, as shown in **Figure 8**, the four detected peaks with m/z 401, m/z 329, m/z 257, and m/z 185 correspond to di, tri, tetra-, and penta-lactate compounds, respectively.

**Table:** 

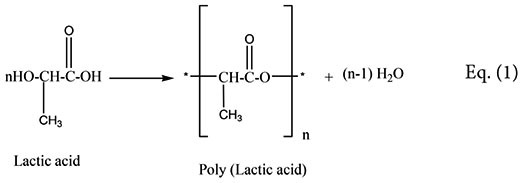

The analysis of the compound DOA14-1 was performed using the Waters Q-TOF II Mass Spectrometer, an LC-MS/MS system that incorporates a quadrupole with the high efficiency of an orthogonal time-of-flight analyzer. Accurate masses of the product ions were measured by the Waters Q-TOF II (**Table 1**). The spectra obtained through this analysis and the accurate mass measurements allowed the identification of the compound DOA14-1 also as a poly (lactic acid) (*n* = 1–4) (**Figure 8**).

**Table 1:** Comparison of the results of accurate mass determinations by Waters Q-TOF 2 Mass Spectrometer and the masses calculated for the ions of cationized DOA14-1 compound.

	Ion (m/z)	Ion formula	Measured mass	Calculated mass	Relative error (ppm)
**[4M-4H+5Na]^+^**	471	C_12_ H_20_ O_12_ Na_5_	471.0443	471.04432	0
**[3M-3H+4Na]^+^**	359	C_9_ H_15_ O_9_ Na_4_	359.0315	359.03069	2
**[2M-2H+3Na]^+^**	247	C_6_ H_10_ O_6_ Na_3_	247.0180	247.01705	4
**[M_2_-H+2Na]^+^**	207	C_6_ H_9_ O_5_ Na_2_	207.0264	207.02454	9
**[M_2_+Na]^+^**	185	C_6_ H_10_ O_5_ Na	185.0443	185.04259	9
**[M-H+2Na]^+^**	135	C_3_ H_5_ O_3_ Na_2_	135.0046	135.00341	9
**[M+Na]^+^**	113	C_3_ H_6_ O_3_ Na	135.0046	113.02146	0

## Discussion

Several studies reported the ability of lactic acid bacteria for production of proteins or proteinaceous compounds as anti-fungal agents (Magnusson and Schnürer, 2001; De Muynck et al., 2004; Falguni et al., 2010). For that purpose, the first extraction experiments from *L. harbinensis K.V9.3.1Np*-mediated fermented yogurt supernatant, consisted in protein precipitation and centrifugal filtration for protein, concentration. In order to minimize the mass effect of casein, we proceeded to a gradual precipitation with ammonium sulfate ranging from 10 to 100% saturation, followed by a separation with a centricon of a 10 kDa cutoff to obtain 20 final proteinaceous fractions. Those fractions were tested for their antifungal potential. Our results revealed the absence of antifungal compounds of proteinaceous nature.

The incorporation of weak acids in industrial food and beverage production for shelf-life increase is a common practice (Huang et al., 2010). Benzoic acid is one of the most commonly acid preservatives used in food for preventing microorganism growth. Benzoic acid produced by *L. plantarum* VTTE-78076 isolated from beer (Niku-Paavola et al., 1999) as well as from *L. plantarum* MiLAB 393 isolated from grass silage (Broberg et al., 2007) were also reported for their antifungal properties. The origin of benzoic acid is not fully elucidated. Lactic bacteria convert hippuric acid which is naturally present in milk into benzoic acid via hippurate hydrolase [EC:3.5.1.32] (phenylalanine degradation) (Sieber et al., 1995). Also, benzoic acid can be produced by auto-oxidation of benzaldehyde (Sieber et al., 1995; Urbienë and Leskauskaitë, 2006) and β-oxidation in the catabolism of fatty acids (Hertweck et al., 2001). Hence, analysis of the available genomes of the starter bacteria *S. thermophilus* JIM 8232 (SAMEA2272807, PRJEA68521, and GCF_000253395.1), *L. plantarum* strains WCFS1, and 5-2 (SAMEA3138345, PRJNA356, GCA_000203855.3, SAMN02953961, PRJNA257680, and GCA_001278015.1) and *L. harbinensis* DSM16991 (SAMN02440850, PRJNA188920, and GCA_000425885.1), showed the absence of several of the enzymes involved in these pathways (data not shown). Further genomic analyses will give us a better understanding of the genetic features and possible pathways responsible for the benzoic acid production or transformation by lactobacilli. Organic acids generated during *L. harbinensis K.V9.3.1Np* growth were previously reported to play an important role in antagonism toward molds (Belguesmia et al., 2014). The implication of weak organic acids in disruption of membrane organization and oxidative stress was supported in several studies (Hazan et al., 2004).

Spermidine and spermine are biogenic polyamine known as an essential polycationic compounds found in all living organisms (Kwon and Lu, 2006). Spermine, widely spread in human and animal tissues was historically reported for its antibacterial activity against a wide range of microorganisms (Fair and Wehner, 1971). However, it was previously reported that due to the predominance of spermidine in fungi, spermidine synthase inhibition or spermidine function alteration may cause fungal growth decrease (Mackintosh et al., 1997). Some spermidine analogs were proved to reduce infection of barley, bean and apple seedlings by a variety of powdery mildew fungus (Mackintosh et al., 1997). Further investigation should be performed to understand the mechanism of action of those new molecules as antifungal compounds but this is the first report on molecules from the spermine family acting as antifungal compounds and produced by bacteria.

Polylactic acid belonging to the class of aliphatic polyesters are known as a biodegradable polymer obtained through the condensation and polymerization of a monomer, lactic acid, that can be derived through the fermentation of carbohydrate feedstock. PLA is well known for its antimicrobial potential used in oligomers solution or associated to organic acids and other antimicrobial agents (Liu et al., 2007). PLA matrix is frequently used in addition to other compounds like pectin (Liu et al., 2007), nisin (Jin and Zhang, 2008), cellulose nanocrystals and silver nanoparticles (Fortunati et al., 2012) and many others for antimicrobial food packaging (Tawakkal et al., 2014).

## Conclusion

In this study, new types of antifungal compounds produced by *L. harbinensis K.V9.3.1Np* were characterized. The ability of extracting, purifying and identifying those compounds was clearly demonstrated. Although the new compounds described in the current work have promising antifungal activities, deeper investigation should be performed to assess their production pathways, mode of action (either acting alone or in synergistic manner) and how they mediate the overall antifungal activity of *L. harbinensis* K.V9.3.1Np Of particular interest, short polylactates, present an interesting potential application in food, feed as well as pharmaceutical industries.

## Author Contributions

AM contributed to study conception, extraction, purification of compounds, data acquisition, analysis and interpretation, and manuscript drafting. ED contributed to data acquisition (antifungal test), analysis, and interpretation. YS contributed to MS spectra analysis and critical revision of the manuscript. MM performed bioinformatics and annotation analysis. PJ and NLY acquired the data. AC interpreted the results and critically revised the manuscript. AB contributed to NMR spectra acquisition and revision of the manuscript. JM conceived the study and revised the manuscript. MB-F contributed to study. GLB conceived and coordinated the study and revised the manuscript.

## Conflict of Interest Statement

The authors declare that the research was conducted in the absence of any commercial or financial relationships that could be construed as a potential conflict of interest.
